# Music Recognition Algorithm based on T-S Cognitive Neural Network

**DOI:** 10.1515/tnsci-2019-0023

**Published:** 2019-04-25

**Authors:** Fei Yan

**Affiliations:** 1 School of Music, Jiaozuo Teachers College, Jiaozuo, Henan 454000, China

**Keywords:** T-S, cognitive neural network, music recognition

## Abstract

The main task of music recognition is to acquire relevant information of music content through processing and feature extraction of audio signals, and then used for comparison, classification, and automatic recording. The cognitive neural network based on T-S model is used to train the network weights with improved genetic algorithm in the paper. The strategy of membership function parameter adjustment is combined with the combination of momentum method and learning rate adaptive adjustment. The new proposed algorithm can be used in the music recognition algorithm by adding a compensation factor related to the input dimension on the membership degree, and the experimental result of the rule disaster caused by the excessive input dimension shows that the new proposed method can be applied to the music recognition system. At the same time, it shows that the accuracy rate of the recognition network is more accurate than that of the other algorithms, and its robustness is better.

## 1 Introduction

Automatic recognition of music is a new interdisciplinary subject, and it relates to the integration concept of multiple disciplines [1]. The main task of music recognition is to acquire relevant information of music content through processing and feature extraction of audio signals, and then used for comparison, classification, and automatic recording. In this paper, the computer recognition of music is the combination of computer multimedia technology, the related knowledge and technology of signal processing and pattern recognition with the music theory [2]. It simulates the process of music cognition analysis by computer, analyzes and analyzes the performance of music and evaluates the performance of the music.

The study of music recognition began in 1970s. The first identification system appeared in 1975. The system in 1996 has been able to deal with more complex piano works. The music recognition network is usually composed of three parts: preprocessing feature extraction and training recognition network. In the study of artificial neural network (ANN) as recognition network, a lot of progress has been made, but the network connection weight value of ANN is the unknown. It is a typical model of black box learning for internal input and output. The understanding of knowledge stored in the process is a difficult problem, and the weight value is not specific. The fuzzy system is based on the fuzzy set theory created by Zadeh. Its obvious characteristic is that it can express the logic directly and it is conformed to human expression of transcendental knowledge. It has good logical function, but the generation and adjustment of membership functions and rules is the most difficult problem. The FNN algorithm synthesized by the neural network and the fuzzy set concept. It can not only imitate the logical idea of the human brain, but also have the capability of the ANN to handle the quantitative and qualitative knowledge synchronously [3,4]. At present, the most commonly used fuzzy neural network model is Takagi-Sugeno (T-S) model[5].

Cognitive style is the personal preference of people in the way of information processing. It is the individual difference in the use of brain-based neural structure and mechanism for information processing. It is also the most extensive and profound individual difference in people’s cognition, which is considered as a relatively fixed tendency. Cognitive style often shows different forms according to the content and situation of cognitive activities. Since the concept of “cognitive style” was put forward, the models, definitions and measuring tools of cognitive style have emerged one after another

The fuzzy neural network based on T-S model is used to train the network weights with improved genetic algorithm in the paper. The strategy of membership function parameter adjustment is combined with the combination of momentum method and learning rate adaptive adjustment. The new proposed algorithm can be used in the music recognition algorithm by adding a compensation factor related to the input dimension on the membership degree, and the experimental result of the rule disaster caused by the excessive input dimension shows that the new proposed method can be applied to the music recognition system. At the same time, it shows that the accuracy rate of the recognition network is more accurate than that of the other algorithms, and its robustness is better.

## 2 Music recognition technology

When musicians communicate and create music works, they are faced with the problems of artificial conversion and low efficiency. The rapid development of information science and technology has provided many solutions to such problems. Although there are many recognition schemes for braille music works at present, there are some disadvantages such as low recognition efficiency and insufficient compatibility. In order to avoid relying too much on artificial experience in traditional braille music image feature extraction, a recognition model based on convolutional neural network was proposed and designed through research. After preprocessing the sample data of braille music images, the model can learn the characteristics of music symbols in braille music images through repeated iterative training.

In the traditional braille music recognition method, the work of manual intervention is large. Although some of the algorithms proposed by traditional models can achieve the feature extraction of braille images to some extent, the recognition effect of braille music images with dot as the basic component and difficult to distinguish is not ideal, which is attributed to their weak learning ability and weak adaptability. In contrast, CNN, which has developed rapidly in recent years, has a relatively optimistic effect, showing high recognition accuracy and strong generalization ability.

**Figure 1:**
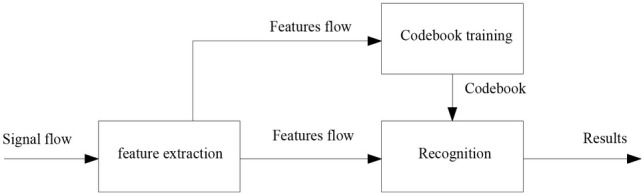
The music recognition flowchart

The main task of music recognition is to use music’s audio signal to get music content, that is to say, get music score. Simply speaking, it is a conversion system from file wav to file. This system has a wide application prospect in the fields of computer music, computer aided composing and music works digitalization, because it can easily realize the MIDI computer entry work of music score. In order to meet the needs of practical applications, music recognition is committed to the recognition of complex music, which contains multiple voices and involves a large number of harmonies. Symphony is a typical representative of this type of Polyphonic Music Recognition type music. Monosyllabic recognition involves only one voice and produces only one note at a time, so its recognition object includes only one note. Moreover, because no harmony is involved, the corresponding note can be easily obtained from the pitch frequency of the signal. The whole recognition task involves only one key technology of pitch frequency extraction. However, because the task of recognition is to get music score, at least two parameters of pitch and pitch of the note should be identified. The music recognition flowchart is shown as Figure 1.

## 3 CNN based on T-S algorithm

### 3.1 CNN structure based on T-S algorithm

Convolution neural network (convolutional neural network, CNN) because of its unique structure, at the same time of image feature extraction, will also be able to extract more details of image information. This not only solves the problem of many parameters and slow training in most traditional neural networks, but also prevents the occurrence of over fitting. Since AlphaGo defeated world go champion lee sedol in 2016, convolutional neural network has been pushed into a wave again, especially in the field of computer vision. Convolutional neural networks have two important characteristics: 1) Shared weights. In the traditional neural network, the weight w of each layer is only used once, and different weight w will be generated when it is used again. However, in the convolutional network, the convolution kernel is convolved with each pixel value (input vector) in the image, so only a set of weights is required. When the input vector is finished with the set of weights, it indicates that the convolution operation is completed. The design of Shared weights does not reduce the time consumption in the forward propagation stage, but to some extent, the number of weight parameters required by the whole model is greatly reduced, which greatly improves the computing performance of the computer. In the operation of convolution, the convolution kernel slides from left to right, from top to bottom on the input image in accordance with the given step size s until the end of the operation. Compared with the traditional neural network, the number of parameters of convolutional neural network is not only reduced, but also its operation speed is improved to a certain extent. 2) sparse connection. In order to mine the information of local association in image space, convolutional neural network adopts the mode of local connection by strengthening the nodes between adjacent layers in the neural network, and abandons the mode of full connection, that is, it adopts the mode of less than the input kernel to complete. For example, if there is a m a n input output, the traditional neural network to each output with each input matrix multiplication, the time complexity, and extraction only meaningful k convolutional neural network input, its time complexity is, because in actual application, general is far less than m, k is more practical significance, and this reduces the time complexity, on the one hand and improve the efficiency of storage.

The CNN algorithm is a decision behavior that uses fuzzy reasoning to imitate human being in the uncertain environment [6]. The

fuzzy rules are constantly adjusted to imitate the system output from the self-training function of the neural network from the initial given fuzzy rules [7, 8, 9]. In the fuzzy algorithm, two main methods are used to express the fuzzy set: one is expressed output set of the fuzzy rules, such as NB, PB, and so on. The other is the formula expression of the fuzzy rule after the input language variable, and the typical case is the linear combination of the input variables. Because the model is first proposed by Takagi and Sugeno, As a result, the T-S algorithm is usually called a fuzzy system.

The convolutional layer is the core structure of the network. Each neuron in this layer is locally connected with the previous layer, and the weight matrix connected is called convolution kernel or filter. The convolution kernel extracts the features of different positions of the input image in the way of “sliding window” with step size s, and outputs a feature graph. A feature graph corresponds to a convolution kernel, that is, the weights of each neuron in the feature graph are Shared. But the features extracted from different feature maps are different. The convolution kernel is the receptive field in the analog visual system, and the size of the convolution kernel

Corresponding to the magnitude of the sensory field, the direction of the convolution kernel corresponds to the direction of the sensory field axon. Different directions of the convolution kernel are used to extract features in different directions, and the first convolution layer is used to extract simple features such as edges or lines. Since the size of the output feature graph decreases with the depth of the convolutional neural network

In the transmission process, the image feature information is not missing, and the number of output feature graph should be increased layer by layer, so that the information in the transmission (the size of output feature graph multiplied by the number) is non-decreasing. Although only simple features are extracted in the low level of the network, with the increase of the network level, each neuron in the high level will feel an increasing number of low-level regions, that is, those simple features extracted in the low level will constantly converge to form complex features in the high level, and finally

obtain the global feature information

Each component *xi* of the input vector *X* is a fuzzy set. The set of linguistic variable values is $T\left(x_{i}\right)=\left\{A_{i}^{1},A_{i}^{2}\cdots A^{m_{i}}\right\},\;\;\;i=1,2,\cdots n$,where $A_{i}^{s_{i}}\left(s_{i}=1,2\cdots m_{i}\right)$is *si* th language variable value of *xi* . Membership function:

$$u_{A_{i}}^{s_{i}}\left(x_{i}\right)\;\;\;\;\left(i=1,2\cdots n;s_{i}=1,2\cdots m_{i}\right)$$

If the output vector is Y, the fuzzy rules of the proposed methods is in the form of (1) $R_{j}:\text{F}\;x_{1}\;\text{is}\;\;A_{1}^{s_{1j}},x_{2}\;\text{is}\;\;A_{2}^{s_{2j}},\cdots ,x_{n}\;\text{is}\;\;A_{n}^{s_{ji}}$

(1) $${\text{THEN y}}_{\text{i}}=w_{j0}+w_{j1}\times x_{1}+w_{j2}\times x_{2}+\cdots w_{ji}\times x_{n}$$

where $j=1,2,\cdots m;m\leq \prod_{i=1}m_{i}.$If the input quantity is fuzzed by the single point fuzzy set, the applicability of each rule can be $a_{j}=u_{A_{1}}^{s_{1j}}\left(x_{1}\right)\;\;\wedge u_{A_{2}}^{s_{2j}}\left(x_{2}\right)\wedge \cdots \wedge u_{A_{n}}^{s_{ji}}\left(x_{n}\right)$the output of the fuzzy system can be written as

(2) $$Y=\sum_{j=1}^{m}a_{j}y_{j}/\sum_{j=1}^{m}a_{j}=\sum_{j=1}^{m}{\bar{a}}_{j}y_{j}\;\;\;\;\;\;\;\;\;\;{\bar{a}}_{j}=a_{j}/\sum_{j=1}^{m}a_{j}$$

**Figure 2:**
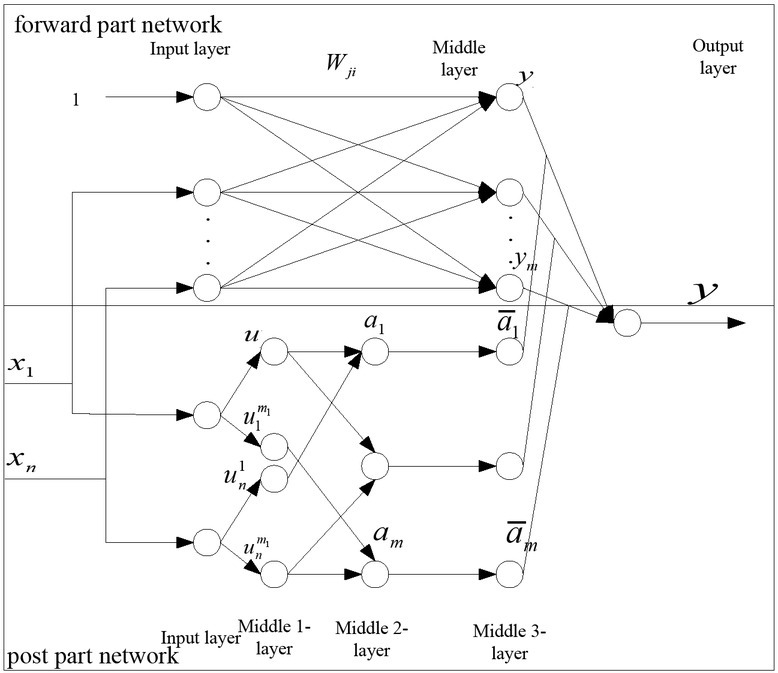
The system diagram of the new proposed algorithm

According to the fuzzy model given above, system diagram of the proposed algorithm is shown in Figure 2. The network is composed of two parts of the forward part network and the post part network. The forward part network is satisfied with the fuzzy rules, and the post part is related to produce the post of the fuzzy rules.

We will analyze each layer of the network, and give the node functions of each layer:

Input layer: Each node is directly connected to each node *xi* , and it plays the role of sending the input information to the next level.

(3) $$f_{i}^{\left(1\right)}=x^{\left(0\right)}=x_{i},\;\;x^{\left(1\right)}=g_{i}^{\left(1\right)}=f_{i}^{\left(1\right)}$$

The input value of the zero nodes in the input layer is

(4) $$x_{0}=1\;,\;\;\;W_{j0},j=1,2,\cdots m\;{\;}_{\cdot }\;\;\text{node :}\;N_{1}=n+1$$

The middle 1-layer of forward part network: Each node represents the value of one language variables. Its effect is to compute membership functions of all input components in fuzzy sets of linguistic variables.

$u_{i}^{s_{i}}\left(x_{i}\right)\;\;\;\;\;\;\;\text{where}u_{i}^{s_{i}}\left(i=1,2\cdots n;s_{i}=1,2\cdots m_{i}\right)$. *m* is the input’ dimensions. *mi* is the fuzzy division number of *si* .

(5) $$N_{2}=\sum_{i=1}^{n}m_{i}$$

(3) The middle 2-layer of forward part network: Its main task is to compute the fitness of each rule.

(6) $$N_{3}=m$$

(4) The middle 3-layer of the forward part

network: The implementation of this level is the normalization calculation.

(7) $$\begin{array}{l} f_{j}^{\left(4\right)}=x_{j}^{\left(3\right)}/\sum_{i=1}^{m}a_{i},x_{j}^{\left(3\right)}={\bar{a}}_{j}=g_{j}^{4}=f_{j}^{\left(4\right)},\\ {\bar{a}}_{j}=a_{j}/\sum_{i=1}^{m}a_{j}\;,\;\;\;N_{4}=m \end{array}$$

(5) The middle layer of the post part network: Each node represents a rule whose action is to express the consequent of formulating rule.

(6) Output layer computing system:

(8) $$Y=\sum_{j=1}^{m}a_{j}y_{j}/\sum_{j=1}^{m}a_{j}=\sum_{j=1}^{m}{\bar{a}}_{j}y_{j}\;\;\;\;\;\;\;\;{\bar{a}}_{j}=a_{j}/\sum_{j=1}^{m}a_{j}$$

It can be seen that *Y* is the cumulating of each rule post, and the weighting coefficient is the normalized applicability. Assuming that the number of fuzzy partitions of each input component is predetermined, the parameter *Wji* to learn is mainly the connection power of the post network. And the central values *Ci*,*s_i_* and widths *ei*,*s_i_* of the membership functions in the middle-layer of the front part are 1. Assuming the error cost function is $E=\frac{1}{2}\left[\bar{Y}-Y\right],\;\;\bar{Y},Y$means the expected output and real output.

### 3.2 Training process of connective parameters based on GA algorithm

In terms of the overall recognition effect, the recognition accuracy of the model trained in this paper is better than most popular methods. Because the braille music picture in this paper is based on the single-sided braille picture, the contrast here is only limited to the recognition and contrast of the single-sided braille picture. The CNN model is compared with feed forward neural network, BP neural network, fuzzy classification algorithm and standard distance positioning method used in recent years, which shows that the recognition accuracy of braille music image in this paper is higher than other methods.

Genetic algorithm is a iterative process. A group of candidate solutions are retained in iterative process. Some iterative operators, such as crossover and mutation, are used to generate a new generation of candidate solutions, which can be repeated to meet some convergence conditions. Genetic algorithms have better robustness. The main feature of genetic algorithms is that genetic algorithms use the encoding of the parameter set rather than the parameters itself; the genetic algorithm aims to guide the genetic process, not to seek derivatives or other theorems; the genetic algorithm searches for optimization in the point group rather than a point; the genetic algorithm uses the possibility transformation. Law, not the finalized law

**Figure 3:**
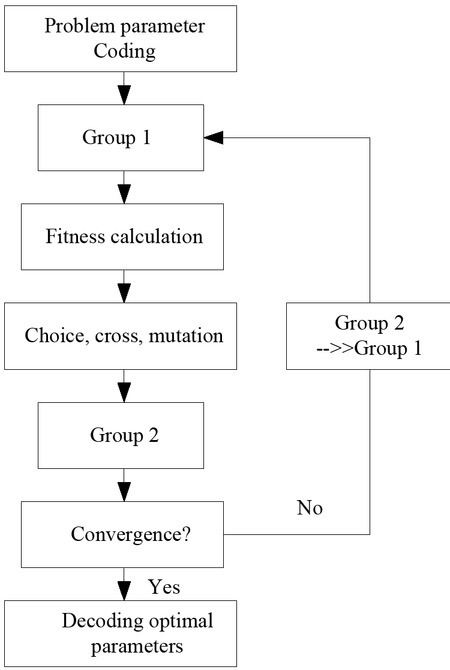
The brief block diagram of genetic algorithm

The training of weights is a complex continuous parameter optimization problem. If binary coding is used, the encoding string will be too long and need to be decoded as real number to make the weight change step into step, thus affecting the accuracy of network learning. The brief block diagram of genetic algorithm is shown in the Figure 3. The optimal weight parameters can be obtained through the flow chart above.

## 4 Simulation experiment and result analysis

### 4.1 Simulation experiment 1

The simulation platform is matlab7.0, and audio samples are from the shocking Music software released by Tsinghua University press in 1997. The sampling rate is 11025 Hz, and the framing standard of the original signal is divided into Hamming windows, 256 frames per frame and 128 frames. In the mixed signal, the voice sample is 42, and the music sample is 67, of which the music sample contains five categories, such as string music, percussion music, woodwind music, brass music and keyboard music features.

At this stage, in order to verify the effectiveness of the convolution neural network recognition model established in the experiment, the initialization of weight parameters in the experiment adopts random Numbers. Secondly, in order to make the model not fall into saturation prematurely during training and affect the learning ability of convolutional neural network, the random number used in the experiment will be relatively small. In this experiment, the training and testing were carried out in a batch-iteration manner, with 200 training iterations respectively, and the accuracy of test data was output every 20 times. Through training and testing, the variation trend of the accuracy is shown in figure 13. Through analysis, it can be seen that after 140 iterations, the accuracy of the model tends to be stable and close to 1. This is because: the structure of music symbol is relatively simple (with dot as the main feature),

and the image resolution used in training and testing is higher, that is, the image quality is better; Secondly, the recognition model is easy to extract the features of braille music symbols in braille music pictures, so that music symbols can be recognized quickly.

The recognition algorithm proposed in this paper compared with the conventional K-means clustering algorithm and short-time energy. The method of fixed clustering center is used in the simulation experiment to fix the clustering center of the mean and variance of the entropy of the phonetic and music samples. The input parameter is the mean and variance of the sample entropy value of each sample, and the output is the class of the sample. The simulation results are compared with the traditional methods of extracting short-time energy and MFCC as features. The results have shown as the above table 1.

**Table 1:** Simulation experimental results

Features	Average recognition rate
T-S Fuzzy neural network	89.073%
Short-time energy algorithm	66.065%
MFCC algorithm	70.642%

### 4.2 Simulation experiment 2

The recognition process is based on the various states that are estimated by the feature flow, and the optimal state sequence is obtained to get the sequence of notes. In view of multiple candidates, the search process should give different length candidates, corresponding to different note detection results. Finally, the recognition results are obtained by multiple candidate decisions.

Recognizing a piece of piano music with 6 notes, the experiment shows that the system can recognize 55 notes correctly; 3 errors are all two consecutive notes with high homophone recognition as one. From hearing, the type error can be tolerated and the recognition rate is 90.2%. By controlling the parameters of the recognition module, this type of error can be avoided, and the recognition rate can be further improved.

For three pieces of music played by piano, Violin and Oboe, the accuracy rate of the new proposed algorithm is shown in the table 2.

**Table 2:** Musical recognition rate of piano, Violin and oboe playing

Timbre	Number of notes	Accurate notes number	Average recognition rate
Piano	61	55	90.2%
Violin	22	22	100%
Oboe playing	58	58	100%
Total	141	135	95.7%

**Figure 4:**
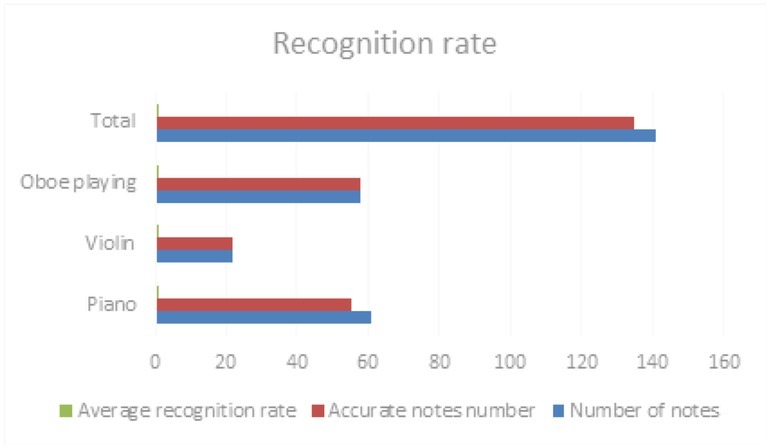
Recognition rate of piano music

As is shown in the Table 2 and Figure 4, the training set is the 61 note played for the piano. It can be seen from this table that the recognition rate is insensitive to both within and outside. The recognition error of piano music is mentioned in the above experimental results; therefore, by adjusting the parameters, the system performance can be further improved. Compared with the recognition rate mentioned above, the performance of the system has been greatly improved. This shows that T-S fuzzy neural network is effective in music recognition.

## 5 Conclusions

The fuzzy neural network based on T-S model is used to train the network weights with improved genetic algorithm in the paper. The strategy of membership function parameter adjustment is combined with the combination of momentum method and learning rate adaptive adjustment. The new proposed algorithm can be used in the music recognition algorithm by adding a compensation factor related to the input dimension on the membership degree, and the experimental result of the rule disaster caused by the excessive input dimension shows that the new proposed method can be applied to the music recognition system. At the same time, it shows that the accuracy rate of the recognition network is more accurate than that of the other algorithms, and its robustness is better.
